# Dichloridobis(3,5-dimethyl-1*H*-pyrazol-4-amine-κ*N* ^2^)cobalt(II)

**DOI:** 10.1107/S1600536808020461

**Published:** 2008-07-09

**Authors:** Xing-Wei Cai, Yu-Yuan Zhao, Guang-Fan Han

**Affiliations:** a School of Materials Science and Engineering, Jiangsu University of Science and Technology, Zhenjiang, Jiangsu 212003, People’s Republic of China

## Abstract

In the title compound, [CoCl_2_(C_5_H_9_N_3_)_2_], the Co^II^ atom adopts a slightly distorted tetra­hedral coordination geometry provided by two chloride anions and two N atoms from the organic ligands. The dihedral angle between the pyrazole rings is 85.91 (10)°. In the crystal structure, mol­ecules are linked into a three-dimensional network by inter­molecular N—H⋯N and N—H⋯Cl hydrogen-bonding inter­actions.

## Related literature

For the crystal structures of related pyrazole compounds, see: Francisco *et al.* (1980 ▶); Murray *et al.* (1988 ▶); Zhao & Eichhorn (2005 ▶).
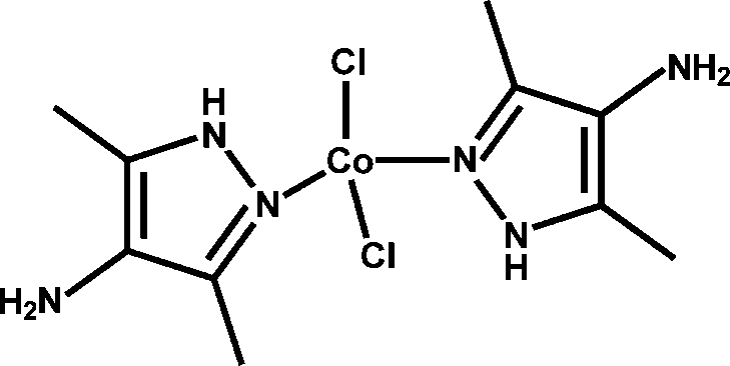


## Experimental

### 

#### Crystal data

[CoCl_2_(C_5_H_9_N_3_)_2_]
*M* *_r_* = 352.13
Triclinic, 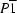

*a* = 9.182 (3) Å
*b* = 9.191 (4) Å
*c* = 10.085 (3) Å
α = 94.807 (13)°
β = 106.105 (4)°
γ = 107.814 (12)°
*V* = 765.1 (5) Å^3^
*Z* = 2
Mo *K*α radiation
μ = 1.47 mm^−1^
*T* = 293 (2) K
0.25 × 0.15 × 0.04 mm

#### Data collection

Rigaku Mercury2 diffractometer
Absorption correction: multi-scan (*CrystalClear*; Rigaku, 2005 ▶) *T*_min_ = 0.836, *T*_max_ = 0.940
7916 measured reflections
3456 independent reflections
2579 reflections with *I* > 2σ(*I*)
*R*_int_ = 0.050

#### Refinement

*R*[*F* ^2^ > 2σ(*F* ^2^)] = 0.043
*wR*(*F* ^2^) = 0.102
*S* = 0.98
3456 reflections
176 parameters
H-atom parameters constrained
Δρ_max_ = 0.35 e Å^−3^
Δρ_min_ = −0.32 e Å^−3^

### 

Data collection: *CrystalClear* (Rigaku, 2005 ▶); cell refinement: *CrystalClear*; data reduction: *CrystalClear*; program(s) used to solve structure: *SHELXS97* (Sheldrick, 2008 ▶); program(s) used to refine structure: *SHELXL97* (Sheldrick, 2008 ▶); molecular graphics: *SHELXTL/PC* (Sheldrick, 2008 ▶); software used to prepare material for publication: *SHELXTL/PC*.

## Supplementary Material

Crystal structure: contains datablocks I, global. DOI: 10.1107/S1600536808020461/rz2230sup1.cif

Structure factors: contains datablocks I. DOI: 10.1107/S1600536808020461/rz2230Isup2.hkl

Additional supplementary materials:  crystallographic information; 3D view; checkCIF report

## Figures and Tables

**Table 1:** Hydrogen-bond geometry (Å, °)

*D*—H⋯*A*	*D*—H	H⋯*A*	*D*⋯*A*	*D*—H⋯*A*
C9—H9*A*⋯Cl1	0.96	2.67	3.570 (5)	157
N2—H2*A*⋯N6^i^	0.86	1.98	2.835 (3)	175
N5—H5*D*⋯N3^ii^	0.86	2.08	2.919 (4)	164
N3—H3*A*⋯Cl2^iii^	0.90	2.56	3.452 (3)	169
N6—H6*B*⋯Cl1^iv^	0.90	2.72	3.457 (3)	140

Symmetry codes: (i) 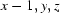; (ii) 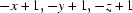; (iii) 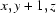; (iv) 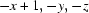.

**Fig. 1.**
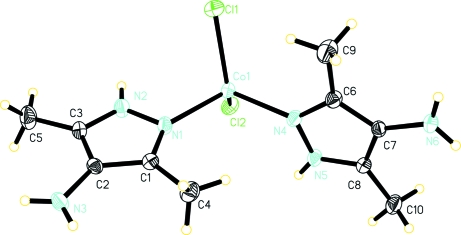
The molecular structure of the title compound with the atom-numbering scheme. Displacement ellipsoids are drawn at the 30% probability level.

## supplementary crystallographic information

### Comment

Pyrazolylmethane late-transition-metal complexes of the first row have shown great potential for the construction of magnetic devices. In the course of our studies of the coordination chemistry of these ligands with cobalt, the title compound was synthesized and we report its crystal structure here.

There have been a few crystal structures reported to date for four-coordinate metal complexes containing two coordinated pyrazoles and two coordinated halides, for examples, dichlorobis(1- phenyl-3,5-dimethylpyrazole)copper(II) (Francisco *et al.*, 1980;), dibromobis(3,5-diphenylpyrazole)copper(II) (Murray *et al.*, 1988) and dichlorobis(3,5-dimethylpyrazole) copper(II) (Zhao & Eichhorn, 2005). The Co—N (2.003 (2) and 2.006 (2) Å) and Co—Cl bond lengths (2.2373 (10) and 2.2829 (11) Å) are within the ranges expected. The dihedral angle formed by the pyrazole rings is 85.91 (10)°. An intramolecular C—H···Cl hydrogen bond (Table 1) helps to stabilzie the molecular conformation. In the crystal structure, molecules are linked by intermolecular N—H···N and N—H···Cl hydrogen bonding interactions to form a three-dimensional network (Table 1).

### Experimental

3,5-Dimethyl-1*H*-pyrazol-4-amine (0.111 g, 1 mmol) was dissolved in ethanol (5 ml) and CoCl_2_ (0.127 g, 1 mmol) in aqueous solution (5 ml) was added with stirring. Colourless crystals suitable for X-ray analysis were obtained by slow evaporation at room temperature over several days

### Refinement

All H atoms were located in a difference Fourier map and refined using the riding-atom approximation, with  C—H = 0.96 Å, N—H = 0.86-0.90 Å, and with *U*_iso_(H) = 1.2 U_eq_ (N) or 1.5 U_eq_ (C).

### Crystal data

**Table:**

[CoCl_2_(C_5_H_9_N_3_)_2_]	*Z* = 2
*M**_r_* = 352.13	*F*_000_ = 362
Triclinic, *P*1	*D*_x_ = 1.528 Mg m^−^^3^
Hall symbol: -P 1	Mo *K*α radiation λ = 0.71073 Å
*a* = 9.182 (3) Å	Cell parameters from 2030 reflections
*b* = 9.191 (4) Å	θ = 2.7–27.5º
*c* = 10.085 (3) Å	µ = 1.47 mm^−^^1^
α = 94.807 (13)º	*T* = 293 (2) K
β = 106.105 (4)º	Plate, colourless
γ = 107.814 (12)º	0.25 × 0.15 × 0.04 mm
*V* = 765.1 (5) Å^3^	

### Data collection

**Table:**

Rigaku Mercury2 (2x2 bin mode) diffractometer	3456 independent reflections
Radiation source: fine-focus sealed tube	2579 reflections with *I* > 2σ(*I*)
Monochromator: graphite	*R*_int_ = 0.050
Detector resolution: 13.6612 pixels mm^-1^	θ_max_ = 27.5º
*T* = 293(2) K	θ_min_ = 2.8º
CCD_Profile_fitting scans	*h* = −11→11
Absorption correction: multi-scan(CrystalClear; Rigaku, 2005)	*k* = −11→11
*T*_min_ = 0.836, *T*_max_ = 0.940	*l* = −13→13
7916 measured reflections	

### Refinement

**Table:**

Refinement on *F*^2^	Secondary atom site location: difference Fourier map
Least-squares matrix: full	Hydrogen site location: inferred from neighbouring sites
*R*[*F*^2^ > 2σ(*F*^2^)] = 0.043	H-atom parameters constrained
*wR*(*F*^2^) = 0.102	*w* = 1/[σ^2^(*F*_o_^2^) + (0.0471*P*)^2^] where *P* = (*F*_o_^2^ + 2*F*_c_^2^)/3
*S* = 0.98	(Δ/σ)_max_ < 0.001
3456 reflections	Δρ_max_ = 0.35 e Å^−^^3^
176 parameters	Δρ_min_ = −0.32 e Å^−^^3^
Primary atom site location: structure-invariant direct methods	Extinction correction: none

### Special details

**Table:**

Geometry. All e.s.d.'s (except the e.s.d. in the dihedral angle between two l.s. planes) are estimated using the full covariance matrix. The cell e.s.d.'s are taken into account individually in the estimation of e.s.d.'s in distances, angles and torsion angles; correlations between e.s.d.'s in cell parameters are only used when they are defined by crystal symmetry. An approximate (isotropic) treatment of cell e.s.d.'s is used for estimating e.s.d.'s involving l.s. planes.
Refinement. Refinement of *F*^2^ against ALL reflections. The weighted *R*-factor *wR* and goodness of fit *S* are based on *F*^2^, conventional *R*-factors *R* are based on *F*, with *F* set to zero for negative *F*^2^. The threshold expression of *F*^2^ > σ(*F*^2^) is used only for calculating *R*-factors(gt) *etc*. and is not relevant to the choice of reflections for refinement. *R*-factors based on *F*^2^ are statistically about twice as large as those based on *F*, and *R*- factors based on ALL data will be even larger.

### Fractional atomic coordinates and isotropic or equivalent isotropic displacement parameters (Å^2^)

**Table:**

	*x*	*y*	*z*	*U*_iso_*/*U*_eq_	
Co1	0.36013 (4)	0.23415 (4)	0.15884 (4)	0.03042 (13)	
Cl1	0.21996 (10)	0.22767 (10)	−0.06317 (8)	0.0469 (2)	
Cl2	0.24095 (9)	0.03437 (9)	0.25653 (8)	0.0412 (2)	
C1	0.4561 (3)	0.5640 (3)	0.3357 (3)	0.0286 (6)	
C2	0.3704 (3)	0.6573 (3)	0.3698 (3)	0.0287 (6)	
C3	0.2086 (4)	0.5675 (3)	0.3138 (3)	0.0343 (7)	
C4	0.6339 (4)	0.6063 (4)	0.3676 (3)	0.0423 (8)	
H4A	0.6603	0.5134	0.3594	0.064*	
H4B	0.6879	0.6646	0.4615	0.064*	
H4C	0.6681	0.6683	0.3025	0.064*	
C5	0.0604 (4)	0.6012 (4)	0.3138 (4)	0.0548 (10)	
H5A	−0.0320	0.5235	0.2461	0.082*	
H5B	0.0687	0.7017	0.2900	0.082*	
H5C	0.0486	0.6001	0.4054	0.082*	
C6	0.6694 (3)	0.2278 (3)	0.0980 (3)	0.0328 (6)	
C7	0.7967 (3)	0.1765 (3)	0.1557 (3)	0.0289 (6)	
C8	0.7838 (3)	0.1407 (3)	0.2824 (3)	0.0322 (6)	
C9	0.6290 (5)	0.2806 (5)	−0.0382 (4)	0.0587 (10)	
H9A	0.5146	0.2584	−0.0741	0.088*	
H9B	0.6621	0.2270	−0.1039	0.088*	
H9C	0.6841	0.3905	−0.0249	0.088*	
C10	0.8851 (4)	0.0783 (4)	0.3884 (3)	0.0491 (9)	
H10A	0.8390	0.0580	0.4624	0.074*	
H10B	0.9923	0.1532	0.4267	0.074*	
H10C	0.8894	−0.0164	0.3445	0.074*	
N1	0.3517 (3)	0.4248 (3)	0.2644 (2)	0.0325 (5)	
N2	0.2026 (3)	0.4300 (3)	0.2516 (3)	0.0382 (6)	
H2A	0.1144	0.3540	0.2085	0.046*	
N3	0.4325 (3)	0.8117 (3)	0.4457 (3)	0.0368 (6)	
H3A	0.3934	0.8723	0.3907	0.044*	
H3B	0.5407	0.8473	0.4675	0.044*	
N4	0.5806 (3)	0.2228 (3)	0.1851 (2)	0.0335 (6)	
N5	0.6537 (3)	0.1685 (3)	0.2974 (2)	0.0342 (6)	
H5D	0.6207	0.1541	0.3687	0.041*	
N6	0.9218 (3)	0.1729 (3)	0.1011 (3)	0.0362 (6)	
H6A	0.8924	0.1782	0.0093	0.043*	
H6B	0.9413	0.0835	0.1104	0.043*	

### Atomic displacement parameters (Å^2^)

**Table:**

	*U*^11^	*U*^22^	*U*^33^	*U*^12^	*U*^13^	*U*^23^
Co1	0.0280 (2)	0.0327 (2)	0.0333 (2)	0.01479 (17)	0.00943 (16)	0.00507 (16)
Cl1	0.0451 (5)	0.0591 (5)	0.0349 (4)	0.0222 (4)	0.0057 (3)	0.0085 (4)
Cl2	0.0346 (4)	0.0415 (4)	0.0474 (5)	0.0122 (3)	0.0119 (3)	0.0154 (3)
C1	0.0293 (15)	0.0267 (15)	0.0297 (15)	0.0091 (11)	0.0094 (11)	0.0068 (11)
C2	0.0342 (16)	0.0288 (15)	0.0249 (14)	0.0114 (12)	0.0116 (11)	0.0057 (11)
C3	0.0357 (17)	0.0332 (17)	0.0374 (17)	0.0173 (13)	0.0117 (13)	0.0038 (13)
C4	0.0331 (17)	0.0422 (19)	0.049 (2)	0.0115 (14)	0.0106 (14)	0.0073 (15)
C5	0.039 (2)	0.048 (2)	0.078 (3)	0.0178 (16)	0.0199 (18)	−0.0025 (18)
C6	0.0285 (16)	0.0401 (17)	0.0332 (16)	0.0120 (12)	0.0133 (12)	0.0117 (13)
C7	0.0249 (14)	0.0283 (15)	0.0322 (15)	0.0085 (11)	0.0090 (11)	0.0005 (11)
C8	0.0262 (15)	0.0392 (17)	0.0314 (16)	0.0140 (12)	0.0075 (11)	0.0031 (12)
C9	0.056 (2)	0.096 (3)	0.053 (2)	0.045 (2)	0.0306 (18)	0.044 (2)
C10	0.049 (2)	0.070 (3)	0.0416 (19)	0.0387 (18)	0.0132 (15)	0.0163 (17)
N1	0.0281 (13)	0.0320 (14)	0.0392 (14)	0.0123 (10)	0.0121 (10)	0.0027 (11)
N2	0.0237 (13)	0.0328 (14)	0.0517 (17)	0.0064 (10)	0.0092 (11)	−0.0040 (12)
N3	0.0401 (15)	0.0314 (14)	0.0364 (14)	0.0109 (11)	0.0113 (11)	0.0016 (11)
N4	0.0322 (14)	0.0442 (15)	0.0313 (14)	0.0198 (11)	0.0122 (10)	0.0122 (11)
N5	0.0347 (14)	0.0486 (16)	0.0309 (13)	0.0232 (12)	0.0165 (10)	0.0146 (11)
N6	0.0297 (14)	0.0422 (15)	0.0398 (15)	0.0145 (11)	0.0148 (11)	0.0033 (11)

### Geometric parameters (Å, °)

**Table:**

Co1—N4	2.003 (2)	C6—C9	1.483 (4)
Co1—N1	2.006 (2)	C7—C8	1.373 (4)
Co1—Cl1	2.2373 (10)	C7—N6	1.412 (3)
Co1—Cl2	2.2829 (11)	C8—N5	1.340 (3)
C1—N1	1.337 (3)	C8—C10	1.488 (4)
C1—C2	1.409 (4)	C9—H9A	0.9600
C1—C4	1.490 (4)	C9—H9B	0.9600
C2—C3	1.384 (4)	C9—H9C	0.9600
C2—N3	1.416 (3)	C10—H10A	0.9600
C3—N2	1.341 (4)	C10—H10B	0.9600
C3—C5	1.486 (4)	C10—H10C	0.9600
C4—H4A	0.9600	N1—N2	1.355 (3)
C4—H4B	0.9600	N2—H2A	0.8600
C4—H4C	0.9600	N3—H3A	0.9000
C5—H5A	0.9600	N3—H3B	0.9000
C5—H5B	0.9600	N4—N5	1.364 (3)
C5—H5C	0.9600	N5—H5D	0.8600
C6—N4	1.349 (3)	N6—H6A	0.9001
C6—C7	1.391 (4)	N6—H6B	0.9000
			
N4—Co1—N1	116.07 (10)	N5—C8—C7	107.2 (2)
N4—Co1—Cl1	114.54 (7)	N5—C8—C10	122.7 (3)
N1—Co1—Cl1	103.32 (8)	C7—C8—C10	130.0 (3)
N4—Co1—Cl2	103.72 (7)	C6—C9—H9A	109.5
N1—Co1—Cl2	104.88 (8)	C6—C9—H9B	109.5
Cl1—Co1—Cl2	114.26 (4)	H9A—C9—H9B	109.5
N1—C1—C2	109.4 (2)	C6—C9—H9C	109.5
N1—C1—C4	122.5 (2)	H9A—C9—H9C	109.5
C2—C1—C4	128.1 (3)	H9B—C9—H9C	109.5
C3—C2—C1	106.1 (2)	C8—C10—H10A	109.5
C3—C2—N3	125.5 (2)	C8—C10—H10B	109.5
C1—C2—N3	128.4 (3)	H10A—C10—H10B	109.5
N2—C3—C2	106.3 (2)	C8—C10—H10C	109.5
N2—C3—C5	122.1 (3)	H10A—C10—H10C	109.5
C2—C3—C5	131.6 (3)	H10B—C10—H10C	109.5
C1—C4—H4A	109.5	C1—N1—N2	106.1 (2)
C1—C4—H4B	109.5	C1—N1—Co1	137.05 (19)
H4A—C4—H4B	109.5	N2—N1—Co1	116.27 (17)
C1—C4—H4C	109.5	C3—N2—N1	112.1 (2)
H4A—C4—H4C	109.5	C3—N2—H2A	124.0
H4B—C4—H4C	109.5	N1—N2—H2A	124.0
C3—C5—H5A	109.5	C2—N3—H3A	109.0
C3—C5—H5B	109.5	C2—N3—H3B	109.1
H5A—C5—H5B	109.5	H3A—N3—H3B	108.0
C3—C5—H5C	109.5	C6—N4—N5	105.4 (2)
H5A—C5—H5C	109.5	C6—N4—Co1	132.8 (2)
H5B—C5—H5C	109.5	N5—N4—Co1	120.26 (18)
N4—C6—C7	109.9 (3)	C8—N5—N4	111.3 (2)
N4—C6—C9	122.4 (3)	C8—N5—H5D	124.3
C7—C6—C9	127.7 (3)	N4—N5—H5D	124.3
C8—C7—C6	106.2 (2)	C7—N6—H6A	109.9
C8—C7—N6	126.4 (3)	C7—N6—H6B	109.9
C6—C7—N6	127.3 (3)	H6A—N6—H6B	108.5

### Hydrogen-bond geometry (Å, °)

**Table:**

*D*—H···*A*	*D*—H	H···*A*	*D*···*A*	*D*—H···*A*
C9—H9A···Cl1	0.96	2.67	3.570 (5)	157
N2—H2A···N6^i^	0.86	1.98	2.835 (3)	175
N5—H5D···N3^ii^	0.86	2.08	2.919 (4)	164
N3—H3A···Cl2^iii^	0.90	2.56	3.452 (3)	169
N6—H6B···Cl1^iv^	0.90	2.72	3.457 (3)	140

Symmetry codes: (i) *x*−1, *y*, *z*; (ii) −*x*+1, −*y*+1, −*z*+1; (iii) *x*, *y*+1, *z*; (iv) −*x*+1, −*y*, −*z*.
